# A European Observational Study to Evaluate the Safety and the Effectiveness of Safinamide in Routine Clinical Practice: The SYNAPSES Trial^1^

**DOI:** 10.3233/JPD-202224

**Published:** 2021-02-02

**Authors:** Giovanni Abbruzzese, Jaime Kulisevsky, Bruno Bergmans, Juan C. Gomez-Esteban, Georg Kägi, Jason Raw, Alessandro Stefani, Tobias Warnecke, Wolfgang H. Jost, the SYNAPSES Study Investigators Group

**Affiliations:** a DINOGMI, University of Genoa, Genova, Italy; b Sant Pau Hospital, Universitat Autònoma de Barcelona, CIBERNED, Barcelona, Spain; c Department of Neurology, AZ St-Jan Brugge-Oostende AV, Campus Brugge, Brugge & Ghent University Hospital, Ghent, Belgium; d Biocruces Research Institute, Barakaldo, Spain; e Department of Neurology, Cantonal Hospital St.Gallen, Switzerland; f Fairfield General Hospital, Bury, Greater Manchester, UK; g Parkinson Center, University Policlinico Tor Vergata, Roma, Italy; h Department of Neurology, University of Muenster, Muenster, Germany; i Parkinson-Klinik Ortenau, University of Freiburg, Wolfach, Germany

**Keywords:** Parkinson’s disease, safinamide, MAO-B inhibitor, real-life evaluation

## Abstract

**Background:**

Safinamide modulates both dopaminergic and glutamatergic systems with positive effects on motor and non-motor symptoms of Parkinson’s disease (PD). The drug utilization study SYNAPSES was designed to investigate the use of safinamide in routine clinical practice, as recommended by the European Medicines Agency.

**Objective:**

To describe the occurrence of adverse events in PD patients treated with safinamide in real-life conditions.

**Methods:**

The SYNAPSES trial is an observational, European, multicenter, retrospective-prospective cohort study. Patients were followed up to 12 months with analyses performed in the overall population and in patients aged >75 years, with relevant comorbidities and with psychiatric conditions.

**Results:**

Of the 1610 patients included, 82.4% were evaluable after 12 months with 25.1% of patients >75 years, 70.8% with relevant comorbidities and 42.4% with psychiatric conditions. During observation 45.8% patients experienced adverse events, 27.7% patients had adverse drug reactions and 9.2% patients had serious adverse events. The adverse events were those already described in the patients’ information leaflet. The majority were mild or moderate and completely resolved and no differences were detected between the subgroup of patients. Clinically significant improvements were seen in the UPDRS motor score and in the UPDRS total score in ≥40% of patients, according to the criteria developed by Shulman et al.

**Conclusion:**

The SYNAPSES study confirms the good safety profile of safinamide even in special groups of patients. Motor complications and motor scores improved with clinically significant results in the UPDRS scale maintained in the long-term.

## INTRODUCTION

Parkinson’s disease (PD) is the second most com-mon neurodegenerative illness following Alzhei-mer’s disease, characterized by the loss of neuro-melanin— containing neurons in the substantia nigra. Approximately 1.2 million people live with PD throughout Europe and the number is expected to double in the next 10 years [1].

The main motor symptoms of PD are resting tremor, bradykinesia and rigidity. The disease is also associated with non– motor symptoms such as depression, apathy, sleep disorders, pain and gastrointestinal disturbances, with a considerable reduction of patients’ quality of life [2]. The current pharmacological management is largely based on symptomatic drugs. Traditional pharmacotherapies for PD aim to restore depleted dopamine levels in the brain but are limited by long-term complications, such as motor fluctuations and dyskinesia. Moreover, the existing medications usually do not alleviate non-motor symptoms [3]. Other neurotransmitters beyond dopamine, in particular glutamate, are believed to play important roles in the pathogenesis of primary symptoms, motor fluctuations, dyskinesia and possibly neuronal cell loss [4].

Safinamide is a multimodal drug with a dual mechanism of action, dopaminergic (reversible mono-amine oxidase-B inhibition) and non-dopaminergic (modulation of the abnormal glutamate release), that offers an innovative approach to the management of motor and non-motor symptoms and motor complications [5]. None of the drugs for PD already on the market have this peculiar double mechanism of action, therefore the Movement Disorder Society has included safinamide in a class of drugs different from selegiline and rasagiline [6].

Despite that the international guidelines recommend double-blind, placebo or active-controlled studies to assess the efficacy and safety of a new therapeutic treatment, they do not provide information on the “real-word” clinical use of these drugs. Many regulatory authorities are thus encouraging the generation of “real-world data” that offer the possibility to derive novel insights on the use and performance of medicines in everyday clinical use, complementing with evidence from randomized control trials [7].

During the initial marketing authorization procedure, the European Medicines Agency (EMA) recommended to provide additional real-world data on safinamide treatment in some categories of patients not well represented in clinical trials, namely those aged >75, with relevant comorbidities and with concomitant psychiatric conditions such as psychosis, bipolar disorder and severe depression. Following this request, a Drug Utilization Study (DUS) called “SYNAPSES” (“european multicenter retrospective-prospective cohort StudY to observe safiNAmide safety profile and pattern of use in clinical Practice during the firSt post-commErcialization phaSe”) was designed to investigate how safinamide is prescribed and used in routine clinical practice and to collect safety data. The aim of the study was merely descriptive and there were no pre-specified hypotheses.

## MATERIALS AND METHODS

### Study design

SYNAPSES (EU PAS Register Number EUPAS-13745) is a multinational, multicenter, retrospective-prospective cohort observational study. The study design was conceived to include potentially all patients treated with safinamide as per clinical practice. The prospective observation was chosen because in most countries the study onset was expected to coincide with the drug commercialization, while the retrospective part was performed to include also patients starting treatment before the study onset. The countries involved were Belgium, Germany, Italy, Spain, Switzerland and United Kingdom. The study was conducted in 128 neurology and geriatric centers, specialized in PD treatment. Physicians participating in the study got an appropriate compensation.

Both the protocol and patient materials were approved by Independent Ethics Committees and Health Authorities in all the participating countries and the study was conducted according to the ethical standards of the institutional and/or national research committee and according to the Declaration of Helsinki.

A total of 1,610 patients were enrolled. This study avoided any selection of patients by means of broad inclusion/exclusion criteria in order to observe the largest population of patients treated with safinamide. Patients were eligible provided they met the following inclusion criteria: male and female patients aged ≥18 years who started treatment with safinamide at the enrolment visit or in the previous four months according to clinical practice, with signed informed and privacy consent forms. Patients were excluded if they were participating in any clinical trial with safinamide at study inclusion. The criteria were more compelling in Germany due to local Health Authority requirements: in compliance with §67 section 6 AMG (German Drug Law) only patients with confirmed diagnosis of PD for whom safinamide was prescribed in accordance with its summary of product characteristics (SmPC) were included, while patients cannot be enrolled in case of contraindications to safinamide as listed in safinamide SmPC. According to the non-interventional study type, the treating physicians were not given any guidelines regarding patient selection and treatment administration and no random procedure was applied. Investigators were asked only to enroll patients in the study consecutively, according to the inclusion/exclusion criteria, provided they were using safinamide in their clinical practice. This was an observational study, hence the physician’s decision of starting treatment with safinamide must be taken before the patient’s inclusion in the study and was completely independent from the study protocol.

As for inclusion criteria, adult patients giving their consent to participate in the study were eligible if they start treatment at the enrolment visit or in the previous four months according to clinical practice. This limit was set in order to allow a reasonable time point for which data are retrospectively collected, leading to the exclusion of a negligible number of patients. All patients were followed for 12 months after the start of treatment. If a patient discontinued treatment with safinamide during the study, the observation continued. Data at baseline (treatment start) and 4, 8, and 12 months after baseline were recorded. Data of patients enrolled at the start of treatment were prospectively collected. Otherwise, data of patients enrolled after the start of treatment were partially retrospective and updated in continuum during the course of the study.

The primary objective of the study was to describe the occurrence of adverse events (AEs) in patients treated with safinamide in real-life conditions for up to one year. The analysis was conducted overall and in the targeted subgroups, namely in patients aged >75, those with relevant comorbidities and those with psychiatric conditions.

The secondary objectives were to describe the characteristics of patients treated with safinamide according to clinical practice and to describe safinamide treatment patterns in real-life settings.

### Data source and measurements

Investigators were asked to record the data in electronic standardized case report forms (eCRFs). Given the observational nature of the study all activities concerning patient management were conducted in compliance with the clinical practice of each center.

For all patients enrolled in the study after the signature of the Informed Consent, data on treatments and on safety outcomes were prospectively recorded by the Investigator both by retrieving those already available from the medical charts and by interviewing the patient. For patients starting treatment with safinamide before the enrolment visit, data were collected retrospectively from each patient’s medical charts at the moment of the enrolment in the study; they were later integrated/updated with information routinely collected interviewing the patient at each subsequent study visit.

The assessments were made in compliance with Good Pharmacoepidemiology Practices and with regulatory and institutional requirements for the protection of confidentiality of patients. Personal data were collected, stored and processed exclusively in pseudonymized form. The eCRF used for the study was validated according to Good Automated Manufacturing Practice version 5 (GAMP5) [8].

Patients were also evaluated with the Unified Parkinson’s Disease Rating Scale (UPDRS) during ON time, measuring the changes from baseline to each follow-up visit in the UPDRS total score and in the UPDRS part III score. The UPDRS [9] was chosen because it is widely known and is the rating scale most commonly used in clinical studies and in routine clinical practice to follow the longitudinal clinical course of PD.

### Statistical methods

The statistical analysis was done on all “evaluable patients for the Full Analysis Set” defined as the patients satisfying all inclusion criteria and not violating any exclusion criteria. All study endpoints were provided using descriptive statistics. In fact, the aim of the study was merely descriptive and there were no pre-specified hypotheses. Categorical variables were described by means of absolute and relative frequencies, while continuous variables by means of mean, standard deviation, quartiles, min and max. Data collected on all patients were pooled for statistical analyses. Stratifications by country were not foreseen because no differences among countries were detected. Patients with missing values were not excluded from the analysis, but their data were not replaced; frequency of missing data was given for all analyzed variables. However, in order to evaluate the potential impact of recall bias on the primary endpoint, the proportion of patients experiencing any AE was provided as sensitivity analysis.

#### Primary objective of the study

The number of patients with AEs (portion of patients experiencing at least one AE from the start of treatment with safinamide until the end of the observation period), serious adverse events (SAEs) and adverse drug reactions (ADRs) related to safinamide, serious or not, were described. The analyses were provided overall and for subgroups of interest: patients aged >75, those with relevant comorbidities and those concomitantly suffering from psychiatric conditions. The concomitant relevant comorbidities (including psychiatric conditions) were those considered by the study Investigators, based only on their own clinical judgement, as clinically significant and/or causing a significant deterioration of patients’ conditions or interfering with PD treatment. Seriousness, severity, relation with safinamide according to Investigator judgment, action taken and outcome of the event were also summarized. Adverse Event terms (AEs, SAEs, and ADRs) were coded with the Medical Dictionary for Regulatory Activities (MedDRA) version 21.1 [10]. Coding was performed at the end of the study and periodically during data collection. Any AE typed in a language different from the one used for encoding (English) was translated and then encoded. At the end of data collection 100% of AEs were reconciled with the Sponsor’s safety database.

#### Secondary objectives of the study

The following endpoints were evaluated:

• Description of demographic and clinical baseline characteristics.
• Description of safinamide treatment duration, including safinamide dose adjustments, inter-ruptions, discontinuation and episodes of overdose.
• Description of changes in other concomitant PD therapies.
• Motor evaluation, as measured by UPDRS III (UPDRS scores was summarized at each time point).

A total of 1,600 patients were expected to be enrolled in the study. This sample size was defined based on safety data available from pivotal clinical trials, where about 66% of patients experienced treatment emergent AEs after 6 months of treatment. With a 20% drop-out rate, 1280 evaluable patients allow to observe the same proportion rate of AEs with a 95% confidence interval between 63% and 68%.

Study results were clinically reviewed and valued by the Study Outcome Review Board. SAS for Windows Version 9.4 and SAS Enterprise Guide 7.1 were used for statistical analyses.

## RESULTS

### Descriptive data

#### Demography

The patients’ overview is shown in Table 1. Out of the 1610 patients enrolled in the SYNAPSES study, 1,558 (96.8%) were evaluable for the analysis, with more than 80% followed up prospectively for one year.

**Table 1:** Patients’ overview

		FAS	Patients aged > 75 y	Patients with relevant comorbidities	Patients with psychiatric conditions
		(*N* = 1,558)	(*N* = 391)	(*N* = 1,103)	(*N* = 661)
Gender (N, %)	Male	961 (61.7%)	221 (56.5%)	665 (60.3%)	356 (53.9%)
	Female	597 (38.3%)	170 (43.5%)	438 (39.7%)	305 (46.1%)
Age at enrolment (y)	Mean (SD)	68.4 (9.7)	79.7 (3.1)	70.0 (8.7)	68.3 (9.4)
Race (N, %)	Caucasian	1,543 (99.0%)	389 (99.5%)	1094 (99.2%)	658 (99.5%)
	Other	15 (1.0%)	2 (0.5%)	9 (0.8%)	3 (0.5%)
Diagnosis (N, %)	Idiopathic PD	1,542 (99.0%)	389 (99.5%)	1094 (99.2%)	653 (98.8%)
	Atypical Parkinsonisms	12 (0.8%)	2 (0.5%)	7 (0.6%)	6 (0.9%)
	Other^*^	4 (0.2%)	0 (0.0%)	2 (0.2%)	2 (0.3%)
Time from PD diagnosis (y): mean (SD)		7.9 (5.3)	7.9 (5.3)	7.8 (5.3)	8.4 (5.5)
Time from PD onset of symptoms (y): mean (SD)		8.8 (5.5)	8.9 (5.5)	8.7 (5.4)	9.3 (5.5)
Age at onset of symptoms (y): mean (SD)		59.3 (11.0)	70.9 (6.5)	61.0 (10.1)	58.8 (10.7)
Hoehn &Yahr stage	1	92 (5.9%)	6 (1.5%)	50 (4.5%)	22 (3.4%)
	2	888 (57.0%)	167 (42.5%)	610 (55.3%)	332 (50,4%)
	3	473 (30.3%)	167 (42.9%)	361 (32.7%)	234 (35.2%)
	4	99 (6.4%)	46 (11.9%)	77 (7.0%)	68 (10.3%)
	5	6 (0,4%)	5 (1.2%)	5 (0.5%)	5 (0.7%)

Percentages (%) were computed by column. FAS, Full Analysis Set; N, number of patients; SD, standard deviation; PD, Parkinson’s disease. ^*^Patients with other diagnosis had juvenile Parkinson’s disease.

Overall, 37.1% of patients had Hoehn and Yahr (H&Y) stage >2 [11]. Lower H&Y stages were observed for younger patients (vs older ones), for patients without (vs patients with) relevant comorbidities and for patients without (vs patients with) psychiatric conditions.

Motor symptoms were reported for 1,151 (99.6%) patients (this information was missing for the remaining 7 patients) and about 88% of subjects had also non-motor symptoms. Tremor and postural instability were more frequent in older patients and in patient with comorbidities, while postural instability and rigidity were preeminent in patients with psychiatric conditions.

Regarding non-motor symptoms, older patients showed a higher frequency of cognitive, cardiovascular, gastrointestinal and urinary symptoms than younger patients. Sleep disorders, psychiatric and cognitive symptoms, fatigue and pain were more frequent in patients with comorbidities and those with psychiatric conditions.

At the start of treatment, the most frequent comor-bidities (>10%) were hypertension and heart diseases, metabolic disorders and joint, bone and pain disorders. The most frequently reported psychiatric conditions (>10%) were depression (mild or moderate) and anxiety (severe depression, bipolar disorders, psychosis and apathy were also present at a lower frequency), with about 28% of patients assuming antidepressants [mainly selective serotonin reuptake inhibitors (SSRIs), serotonin– norepinephrine reuptake inhibitors (SNRIs) and tricyclics].

#### Concomitant PD medications

Almost all patients (*N* = 1,556, 99.9%) had≥1 ongoing treatment for PD at the start of safinamide therapy. As shown in Table 2, 1,537 patients (98.7%) were treated with levodopa (L-dopa), 912 (58.5%) dopamine agonists, 424 (27.2%) catechol-O-methyltransferase (COMT) inhibitors, 178 (11.4%) amantadine, 34 (2.2%) anticholinergics and 11 (0.7%) monoamine oxidase-B (MAO-B) inhibitors (rasagiline). Over the study period, the overall mean dose of levodopa (alone or plus COMT inhibitors) and the overall mean doses of the dopamine-agonist treatments did not change significantly, confirming what has been previously observed in the pivotal trials [12, 13].

**Table 2:** Concomitant PD treatments at the start of safinamide

Categories	Total number of evaluable patients (FAS)
	(*N* = 1,558)
Levodopa	1,537 (98.7%)
Dopamine agonist	912 (58.5%)
COMT inhibitors	424 (27.2%)
Amantadine	178 (11.4%)
Anticholinergics	34 (2.2%)
MAO-B inhibitors	11 (0.7%)

Percentages are computed out of the total number of patients evaluable for FAS. A patient could have more than one PD treatment. PD, Parkinson’s disease; FAS, Full Analysis Set; N, number of patients; COMT, catechol-o-methyltransferase; MAO-B, monoamine-oxidase B.

#### Motor fluctuations

As shown in Table 3, at start of safinamide treatment 1437 (92.2%) patients had motor fluctuations, the most frequent one being wearing off (74.3%).

**Table 3:** Fluctuations at the start of treatment with safinamide and during the follow-up

Motor complications	FAS	Total evaluable patients at 4 months	Total evaluable patients at 8 months	Total evaluable patients at 12 months
	(*N* = 1,558)	(*N* = 1,373)	(*N* = 1,323)	(*N* = 1,326)
Any	1,437 (92.2%)	1,009 (73.5%)	934 (70.6%)	894 (67.4%)
Wearing-off	1,158 (74.3%)	752 (54.8%)	704 (53.2%)	701 (52.9%)
Early morning fluctuations	363 (23.3%)	198 (14.4%)	196 (14.8%)	182 (13.7%)
Unpredictable fluctuations	264 (16.9%)	152 (11.1%)	149 (11.3%)	133 (10.0%)
Delayed on	177 (11.4%)	108 (7.9%)	109 (8.2%)	109 (8.2%)
Dyskinesia	610 (39.2%)	469 (34.2%)	409 (30.9%)	369 (27.8%)
Other	87 (5.6%)	62 (4.5%)	52 (3.9%)	32 (2.4%)

Percentages were computed out of the number of patients evaluable for the FAS and for each follow up visit. FAS, Full Analysis Set; N, number of patients.

The percentage of patients with motor fluctuations decreased during the study due to safinamide treatment: at 4, 8, and 12 months after start of treatment, the percentage of patients with motor fluctuations was respectively 73.5%, 70.6%, and 67.4%, with the highest reduction for wearing-off and early morning fluctuations. The improvements seen after 4 months indicate a rapid onset of efficacy of the drug. The percentage of patients with fluctuations has been calculated at each follow-up visits based on the available data and on the total number of the evaluable patients.

### Primary endpoint

#### Adverse events and serious adverse events

**Fig. 1:**
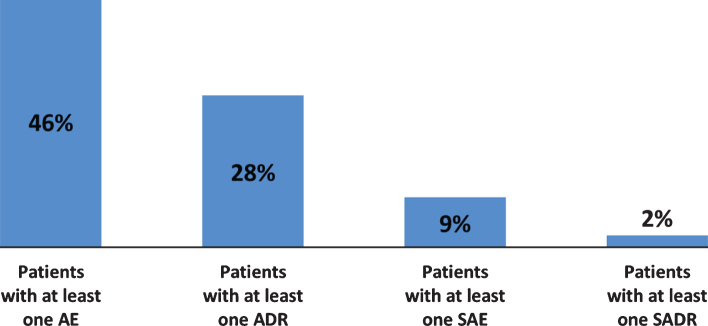
Adverse events and adverse reactions during observation (overall). AE, Adverse Event; SAE, Serious Adverse Event; ADR, Adverse Drug Reaction; SADR, Serious Adverse Drug Reaction.

As reported in Fig. 1, during observation 714 (46%) patients experienced at least one AE and 432 (28%) patients experienced at least one ADR; 143 patients (9%) had at least one SAE and 36 patients (2%) had at least one SADR. The percentage of patients experiencing AEs during one year of treatment with safinamide in real-life conditions was 30% lower compared to the percentage observed in six-months pivotal trials [12, 13].

Adverse events were mainly mild (62.0%) or moderate (28.0%): only 2% were considered to have a definite relationship with safinamide and in the majority of the cases no action was taken regarding the treatment and the outcome was resolved. The monthly incidence rate of AEs was very low, 0.07 AEs per patient per month. In Table 4 the AEs are classified by system organ class (SOC).

**Table 4:** Distribution of adverse events severity by system organ class

System Organ Class	Any	Mild	Moderate	Severe
	*N* = 1,435 (100%)	*N* = 888 (62%)	*N* = 404 (28%)	*N* = 143 (10%)
Nervous system disorders	480 (33.4%)	345 (24.0%)	110 (7.7%)	25 (1.7%)
Psychiatric disorders	205 (14.3%)	121 (8.4%)	68 (4.7%)	16 (1.2%)
Gastrointestinal disorders	107 (7.4%)	73 (5.1%)	29 (2.0%)	5 (0.3%)
Injury, poisoning and procedural complications	99 (6.9%)	36 (2.5%)	41 (2.9%)	22 (1.5%)
Musculoskeletal and connective tissue disorders	94 (6.5%)	55 (3.8%)	37 (2.6%)	2 (0.1%)
General disorders	93 (6.5%)	65 (4.5%)	21 (1.5%)	7 (0.5%)
Infections and infestations	75 (5.2%)	32 (2.2%)	18 (1.3%)	25 (1.7%)
Eye disorders	44 (3.1%)	34 (2.4%)	10 (0.7%)	0 (0%)
Vascular disorders	37 (2.5%)	23 (1.6%)	12 (0.8%)	2 (0.1%)
Respiratory, thoracic and mediastinal disorders	32 (2.3%)	16 (1.1%)	8 (0.6%)	8 (0.6%)
Skin and subcutaneous tissue disorders	27 (1.9%)	22 (1.5%)	4 (0.3%)	1 (0.1%)
Renal and urinary disorders	26 (1.8%)	16 (1.1%)	7 (0.5%)	3 (0.2%)
Cardiac disorders	25 (1.7%)	5 (0.3%)	9 (0.6%)	11 (0.8%)
Metabolism and nutrition disorders	18 (1.2%)	12 (0.8%)	4 (0.3%)	2 (0.1%)
Investigations	12 (0.8%)	9 (0.6%)	3 (0.2%)	0 (0%)
Neoplasms benign, malignant and unspecified	12 (0.9%)	1 (0.1%)	3 (0.2%)	8 (0.6%)
Surgical and medical procedures	12 (0.8%)	3 (0.2%)	7 (0.5%)	2 (0.1%)
Ear and labyrinth disorders	11 (0.8%)	11 (0.8%)	0 (0%)	0 (0%)
Hepatobiliary disorders	7 (0.5%)	0 (0.0%)	6 (0.4%)	1 (0.1%)
Blood and lymphatic system disorders	6 (0.4%)	3 (0.2%)	2 (0.1%)	1 (0.1%)
Reproductive system and breast disorders	5 (0.4%)	3 (0.2%)	1 (0.1%)	1 (0.1%)
Endocrine disorders	2 (0.2%)	1 (0.1%)	1 (0.1%)	0 (0%)

Percentages were computed out of the total number of AEs and data sorted by descending frequency of SOC. N, number of patients.

Dyskinesia was the most frequently reported AE among nervous system disorders, although it occurred in a lower frequency in the SYNAPSES study compared to previous pivotal trials (13.7% vs 18%).

Other AEs with a frequency≤3% of the total number of events were hallucinations (*N* = 41, 2.9%), fall (*N* = 25, 2.3%), nausea (*N* = 18, 1.7%), constipation (*N* = 14, 1.3%) and headache (*N* = 14, 1.3%). The AEs observed were those already described in the patients’ leaflet and no differences were detected in term of nature, frequency, causal relationship or severity.

Forty-four eye disorders (3.1% of the total number of AEs) were observed, all non serious: the most frequent ones (<1%) were blurred vision, reduced visual acuity and diplopia. It must be noted that data on the concomitant use of safinamide in patients with retinopathy of any type are not available because these subjects were not enrolled. Nevertheless, no relevant frequency of cataract was reported thus no signals of ophthalmological relevance were detected.

As previously reported, 194 serious adverse events (SAEs) occurred in about 9% of patients. SAEs were about 14% of all adverse events. The most frequently reported SAEs by system organ class were infections and infestations (*N* = 38, 3.0% of all occurred AEs), injury, poisoning and procedural complications (*N* = 31, 2.0%) and nervous system disorders (*N* = 29, 2.0%).

Among the above, the most frequent were pneumonia (*N* = 9), urinary tract infections (*N* = 6), dyskinesia (*N* = 5) and femur fractures (*N* = 5).

The majority of SAEs was completed resolved. No SAEs had a definite relationship with safinamide and only eight (4.0% of all SAEs) and four (2.0% of all SAEs) had a possible or probable relationship with safinamide, respectively.

Patients with a partially retrospective observation period (having started treatment with safinamide before study inclusion) were 779 (50% of evaluable patients). No differences were detected in term of AE or SAE frequency, based on the sensitivity analysis, between patients who started safinamide before or after study inclusion.

A total of 233 (16.9%) evaluable patients had non-appropriate use of safinamide (e.g., absence of fluctuations, safinamide starting dosage different from 50 mg/day, no concomitant levodopa use, diagnosis different from idiopathic PD or concomitant treatment with other MAO-B inhibitors). These data were captured in the SYNAPSES study because they represent the real-practice of the PD treatment. Although not initially foreseen by the study protocol, a descriptive analysis on the frequency of safety events was provided in patients undergoing appropriate use of safinamide vs inappropriate use: no differences were detected between the two groups.

### Secondary endpoints

#### Safinamide treatment patterns

Safinamide was administered at an initial dose of 50 mg/day to 94% of patients; during the observation period, 58.0% of patients had a dose increase from 50 to 100 mg due to “clinician decision” and 6% a dose decrease from 100 to 50 mg due to “patient choice”. Safinamide temporary discontinuation was observed for 68 patients (4.4%), while 336 patients (21.6%) permanently discontinued the drug (a similar percentage was reported in pivotal trials). Main reasons for discontinuation were adverse events (*N* = 161, 10.3%), patient choice (*N* = 81, 5.1%) and disease progression (*N* = 20, 1.3%). No relevant differences regarding the treatment patterns with safinamide emerged between the subgroup of patients (elderly, with relevant comorbidities and with psychiatric conditions).

#### Subgroup of patients

**Fig. 2:**
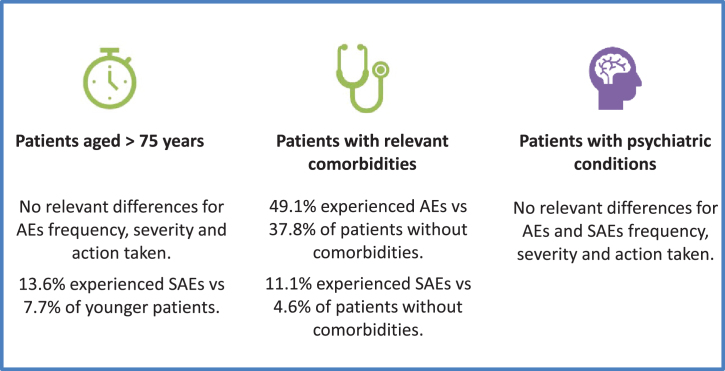
Safety summary in subgroups of patients. AE, Adverse Event; SAE, Serious Adverse Event.

The summary of the safety results of the three subgroup of interest (patients aged >75 years, patients with relevant comorbidities and patients with psychiatric conditions) is shown in Fig. 2.

*Elderly patients.* During observation 47.3% patients older than 75 years had at least one AE, 26.1% at least one ADR, 13.6% at least one SAE and 2.3% at least SADR.

The proportions of patients experiencing at least one AE, ADR or SADR were similar in the population of patients aged >75 compared to patients aged ≤75, while was higher for SAE in patients aged >75 (13.6%) compared to patients aged ≤75 (7.7%). No relevant differences emerged in severity, causal relationship or action taken between the two age groups. Dyskinesia was still the most frequent AE in elderly patients, but with a lower frequency than the overall sample and the younger patients (9.5% vs 13.7%) while hallucinations (5.1% vs 2.9%) were slightly more frequent in patients aged >75.

*Patients with comorbidities.* During observation 49.1% patients with relevant comorbidities had at least one AE, 28.6% had at least one ADR, 11.1% had at least one SAE and 2.5% at least one SADR.

The proportion of patients with AEs and SAEs was higher in patients with relevant comorbidities than in patients without (49.1% vs 37.8% and 11.1% vs 4.6% respectively). No other differences were detected.

Dyskinesia was the most frequent AE in the subgroup of patients with comorbidities, with a slightly lower frequency than the overall sample and the patients without comorbidities (12.3% vs 13.7%). The distribution of the occurrence of the other AEs was similar in the two subgroup of patients (with and without comorbidities) and the overall sample.

*Patients with psychiatric conditions.* During observation, 47.8% patients with psychiatric conditions had at least one AE, 31.0% at least one ADR, 10.3% at least one SAE and 3.5% at least one SADR. No relevant differences were observed in patients with or without psychiatric conditions regarding the severity, causal relationship and action taken for adverse events. No serotoninergic syndromes were reported despite 28% of patients were assuming antidepressant drugs including fluoxetine, thus confirming that the concomitant use of safinamide and SSRIs/SNRIs/tricyclics is safe and well tolerated.

Dyskinesia was the most frequent AE in the subgroup of patients with psychiatric conditions, with a slightly higher frequency than in the overall sample and in patients without psychiatric conditions (15.6% vs 13.7%).

#### UPDRS scores

**Fig. 3:**
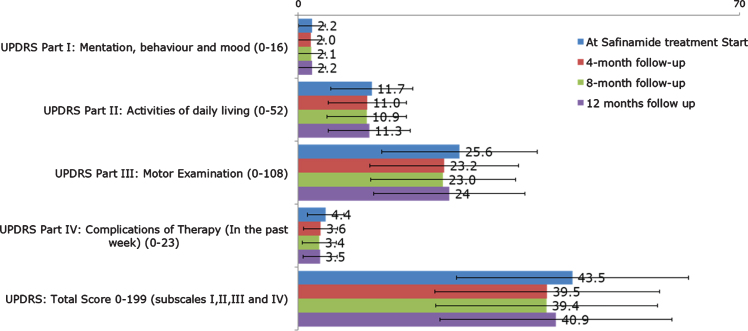
UPDRS (part I, II, III, IV, total scores) at start of treatment with safinamide and at 4-, 8-, and 12-month follow-up (overall). UPDRS, Unified Parkinson’s Disease Rating Scale.

The mean (SD) of UPDRS (part I, II, III, IV, total) scores at the beginning of treatment with safinamide and at 4-, 8-, 12-month follow up are shown in Fig. 3. A substantial stability of disease severity over time emerged with improvements since the first 4-month follow-up in the UPDRS part II (Activities of Daily Living) part III (Motor Examination), part IV (Complication of Therapy) and UPDRS Total Score.

**Fig. 4:**
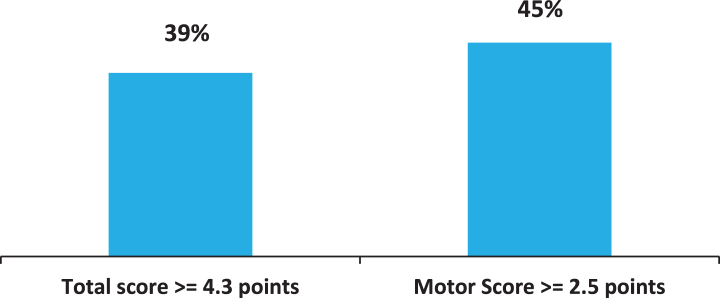
Percentage of patients with clinically important difference (improvement) in the UPDRS scores (difference between 12-months follow-up and baseline). UPDRS, Unified Parkinson’s Disease Rating Scale.

According to the criteria developed by Shulman [14], a difference >4.3 points for the UPDRS Total score (subscales I, II, III and IV) and >2.5 points for the UPDRS Part III (Motor Examination score) is considered clinically significant. The percentage of patients with clinically relevant differences between baseline and 12-month follow up visit is reported in Fig. 4. After one-year treatment with safinamide, 39% and 45% patients showed a clinically significant improvement in UPDRS Total and Motor Examination scores, respectively.

## DISCUSSION

The SYNAPSES study documented for the first time the real-world use of safinamide in six European countries, particularly in patients’ populations such as patients older than 75, with psychiatric illnesses or with relevant comorbidities, where limited information was available in the pre-authorization clinical trials. The results have been analyzed and presented as pooled data of the two safinamide doses, anyway during the study 58% of patients increased the dose of safinamide from 50 to 100 mg with no differences in term of adverse events, including dyskinesia, confirming what has been reported in the literature.

Safinamide was safe and well tolerated and no major or unexpected safety concerns were identified. The percentage of patients experiencing AEs was 30% lower compared to the percentage observed in previous pivotal trials. This is remarkable because pivotal trials have strict inclusion/exclusion criteria and enroll generally patients with less comorbidities than subjects in a practice setting.

The rate of AEs observed with safinamide was also 50% lower than the percentage reported in the same patients’ population for rasagiline in the PRESTO study (45.7% vs 93% respectively) [15] and 40% lower than the percentage reported for opicapone in the OPIPARK study (45.7% vs 74.9% respectively) [16].

The most frequent reported AE was dyskinesia, as expected with drugs that increase levodopa bioavailability [17], although at very lower frequency than those observed in previous pivotal clinical trials. However, it is important to note that most PD patients who complained of dyskinesia have presented these abnormal movements since the beginning of the study with no further aggravation.

The treatment with safinamide did not require any change in the concomitant dopaminergic therapies, allowing a better motor control with no safety problems. Despite its dopaminergic activity, impulse control disorder or sleep deterioration were not reported after safinamide treatment. Safinamide increases dopamine levels in the putamen, the region devoted to motor control, but does not affect regions involved in reward circuitry and impulse control disorders (ICDs) such as the nucleus accumbens [18]. Moreover, glutamate levels seem to mediate reward-seeking behavior within this nucleus [19]. Safinamide is a MAO-B inhibitor and a glutamate modulator and this second mechanism of action, in addition to the non-influence on the mesolimbic structures, may contribute to avoid any ICD deterioration.

At the start of the study about 60% of the patients were receiving dopamine agonists (DA) as concomitant treatment with L-dopa. It is reported in the literature that 15% to 20% of patients on DA exhibit impulse control disorder behavior (e.g., gambling and hypersexuality), imposing a significant economic burden and a quality of life deterioration of patients and their caregivers [20]. The adjunct of safinamide improved the symptoms without tolerability problems and with no need to increase the DA dose or switch to another drug.

Regarding sleep disorders, it is known that dopa-mine agonists alone or combined with levodopa can cause sleep disturbances and episodes of sudden sleep attacks [21]. Glutamate is an important wake-active neurotransmitter producing a sustained behavioral and electro-encephalogram arousal [22]. The absence of sleep disturbances in PD patients taking safinamide may be related to the effects of the drug on the glutamatergic system.

The safety profile of safinamide in patients over 75 years, with comorbidities and with psychiatric conditions appeared to be broadly similar to the overall population. The slightly higher percentage of SAEs observed in elderly patients and in patients with comorbidities is an expected phenomenon since they are generally affected by several relevant concomitant pathologies treated with polytherapies. This is confirmed by the fact that ADRs did not occur more frequently in these patients, suggesting that there were no contraindications to the use of safinamide in these subjects. Safinamide in fact, unlikely rasagiline, is not metabolized by CYP1A2 and thus does not have any important drug-drug interaction. The slightly higher frequency of hallucinations in elderly patients reflects, as known, age as risk factor for hallucinations [23].

The absence of any safety issue in psychiatric patients, despite their concomitant treatment with antidepressant drugs, suggests that safinamide does not require special safety precautions for these subjects. Safinamide, in fact, increases the synaptic availability of dopamine without a direct interaction with dopamine D2 receptors that are known to be involved in psychotic behaviors [18].

Motor fluctuations, and in particular wearing-off and early morning fluctuations, affect the majority of patients with PD: as observed in the DEEP study, 63– 75.6% of patients treated with L-dopa experienced wearing-off with a significant deterioration of their quality of life [24]. The pharmacological treatment of motor fluctuations is difficult and remain a real unmet need [25]. There is a significant association between motor fluctuations and annual costs of PD: the mean costs of patients with motor fluctuations is generally two-three times greater than the costs of patients without [26]. Treatment strategies capable of delaying the onset or attenuating the severity of motor fluctuations could be expected to improve QoL and reduce some of the economic burden of PD. Safinamide was shown to reduce of about 40– 50% motor fluctuations, in particular wearing-off and early morning fluctuations, with visible efficacy already at 4 months. This significant and rapid-onset effect of safinamide may be explained by its dual mechanism of action, dopaminergic and glutamatergic [27]. The pathogenesis of motor fluctuations, in fact, suggests that glutamate and other neurotransmitters, in addition to dopamine, contribute to the appearance of the symptoms [28].

Consistent with the benefits observed in motor fluctuations, UPDRS scores improved with safinamide as adjunct therapy after only 4 months of treatment with a clinically important effect in ≥40% of patients according to the criteria developed by Shulman et al. [14]. In term of absolute values, the magnitude of the effect of safinamide on motor symptoms is similar to that reported for other MAO-B inhibitors and for COMT inhibitors by Stowe et al. [29]. As expected, the improvements are higher at 4 and 8 months due to the progression of the pathology, nevertheless the results obtained for the motor score are noteworthy because the patients were already receiving an optimized dopaminergic therapy (based on clinician’s judgement) and further improvements were unexpected. The annual decline in early untreated PD patients is 5– 6 points for UPDRS III motor score [30]. Dopaminergic therapies reduce the decline in motor function to an annual downfall of 3.3 points [31]. The add-on of safinamide to a standard levodopa therapy compensates this progressive deterioration in 45% of patients allowing for a substantial stability of their motor symptoms.

The main strengths of this study are the large number of fluctuating patients included, with a broad range of disease severity (Hoehn and Yahr stages I– IV), the real-life clinical practice setting and the relevant sample sizes for the subgroups of interest. Limitations include the open-label design without a placebo or active control, typical of the observational studies, where the expectations of the patients are generally higher than in double-blind trials. Patients diaries for ON and OFF time were not adopted, as suggested by EMA, because they are not used in routine clinical practice. There was also a potential risk of under-estimation of AEs from both the patients and the clinicians: however, no relevant difference was observed when considering the frequency of AEs or SAEs in retrospective or prospective patients.

### Conclusions

The management of motor complications in PD remains a significant challenge in which all available pharmacologic options involve a risk of exacerbating adverse events. The SYNAPSES study, conducted in a real word setting in six European countries, confirmed the safety and tolerability of safinamide, as adjunct therapy, in fluctuating patients and in special groups of subjects. Neither age, comorbidities, nor psychiatric conditions seem to have any relevant effect on its safety profile.

Motor complications and motor scores improved with clinically significant results in the UPDRS scale maintained in the long-term. These results suggest that safinamide can be an effective and safe option for the management of motor fluctuations in levodopa-treated patients.

## CONFLICT OF INTEREST

G. Abbruzzese, J. Kulisevsky, W. H. Jost, B. Bergmans, J.C. Gomez-Esteban, G. Kägi, J. Raw, A. Stefani, and T. Warnecke are members of the Scientific Advisory Board of Zambon SpA.

### SYNAPSES Study Investigators Group

Belgium: Bergmans B, Bourgeois P, Cras P, De Klippel N, Dethy S, Franco G, Garraux G, Geens K, Jacquerye P, Jeanjean A, Santens P, Supiot F, Van der Linden C.

Germany: Blersch WK, Delf M, Hellwig B, Herbst HP, Kupsch A, Jost WH, Lang M, Muhlack S, Nastos I, Oehlwein C, Schlegel E, Schwarz J, Warnecke T, Woitalla D.

Italy: Abbruzzese G, Aguggia M, Avarello T, Barone P, Baruffaldi R, Belgrado E, Bentivoglio AR, Bosco D, Calabresi P, Callegarini C, Cannas A, Centonze D, Ceravolo R, Colosimo C, Comi C, Contardi S, Cortelli P, Cossu G, D’Amelio M, De Pandis MF, Denaro A, Di Lazzaro V, Fabbrini G, Gasparoli E, Guidi M, Iliceto G, Lopiano L, Manganotti P, Marconi R, Marini C, Marsala SZ, Mauri M, Moleri M, Monge A, Morgante F, Negrotti A, Nordera G, Onofrj M, Pacchetti C, Padovani A, Pontieri FE, Priori A, Quatrale R, Sensi M, Stefani A, Tamma F, Tessitore A, Tinazzi M, Vitale C, Volontè MA, Zappia M, Zecchinelli AL.

Spain: Arbelo Gonzalez JM, Bayés A, Blazquez M, Calopa Garriga M, Callen A, Campos Arillo V, Cubo E, De Fábregues O, Escalante Arroyo S, Espinosa Rosso R, Esquivel López A, Freire E, García Cobos E, García Moreno JM, Gomez-Esteban JC, González-Ardura J, Grandas Perez F, Kulisevsky J, Kurtis M, Juni J, Legarda I, Leiva C, López Aristegui N, López Manzanares L, Lozano JJ, Luquín MR, Martinez Castrillo JC, Martí Domenech MJ, Martínez I, Mata M, Mir Rivera P, Pascual Sedano B, Rodríguez Oroz MC, Rodríguez Uranga JJ, Sanchez S, Santos García D, Solano B, Vaamonde Gamo J.

Switzerland: Accolla E, Bohlhalter S, Kälin A, Kägi G, Michelis J.

U.K.: Carrol C, Henderson E, Raha S, Raw J, Silva N, Silverdale M.
